# The ENHANCES study—Enhancing Head and Neck Cancer patients’ Experiences of Survivorship: study protocol for a randomized controlled trial

**DOI:** 10.1186/1745-6215-15-191

**Published:** 2014-05-28

**Authors:** Jane Turner, Patsy Yates, Lizbeth Kenny, Louisa G Gordon, Bryan Burmeister, Damien Thomson, Brett Hughes, Alexandra L McCarthy, Chris Perry, Raymond J Chan, Alana Fraser, Helen Skerman, Martin Batstone, Kathryn Carswell

**Affiliations:** 1 School of Medicine, University of Queensland, Herston Rd, Herston, QLD 4029, Australia; 2 Cancer Care Services, Royal Brisbane and Women’s Hospital, Herston Rd, Herston, QLD 4029, Australia; 3 Institute of Health and Biomedical Innovation, Queensland University of Technology, Musk Ave, Kelvin Grove, QLD 4059, Australia; 4 Centre for Applied Health Economics, Griffith Health Institute, Griffith University, University Drive, Meadowbrook, QLD 4131, Australia; 5 Cancer Care Services, Princess Alexandra Hospital, Ipswich Rd, Woolloongabba, QLD 4101, Australia; 6 Cancer Services Southern Clinical Network, Princess Alexandra Hospital, Ipswich Rd, Woolloongabba, QLD 4101, Australia

**Keywords:** Head and neck cancer, Self-management, Survivorship

## Abstract

**Background:**

Few cancers pose greater challenges than head and neck (H&N) cancer. Residual effects following treatment include body image changes, pain, fatigue and difficulties with appetite, swallowing and speech. Depression is a common comorbidity. There is limited evidence about ways to assist patients to achieve optimal adjustment after completion of treatment. In this study, we aim to examine the effectiveness and feasibility of a model of survivorship care to improve the quality of life of patients who have completed treatment for H&N cancer.

**Methods/Design:**

This is a preliminary study in which 120 patients will be recruited. A prospective randomised controlled trial of the H&N Cancer Survivor Self-management Care Plan (HNCP) involving pre- and post-intervention assessments will be used. Consecutive patients who have completed a defined treatment protocol for H&N cancer will be recruited from two large cancer services and randomly allocated to one of three study arms: (1) usual care, (2) information in the form of a written resource or (3) the HNCP delivered by an oncology nurse who has participated in manual-based training and skill development in patient self-management support. The trained nurses will meet patients in a face-to-face interview lasting up to 60 minutes to develop an individualised HNCP, based on principles of chronic disease self-management. Participants will be assessed at baseline, 3 and 6 months. The primary outcome measure is quality of life. The secondary outcome measures include mood, self-efficacy and health-care utilisation. The feasibility of implementing this intervention in routine clinical care will be assessed through semistructured interviews with participating nurses, managers and administrators. Interviews with patients who received the HNCP will explore their perceptions of the HNCP, including factors that assisted them in achieving behavioural change.

**Discussion:**

In this study, we aim to improve the quality of life of a patient population with unique needs by means of a tailored self-management care plan developed upon completion of treatment. Delivery of the intervention by trained oncology nurses is likely to be acceptable to patients and, if successful, will be a model of care that can be implemented for diverse patient populations.

**Trial registration:**

ACTRN12613000542796 (registered on 15 May 2013)

## Background

Historically, the diagnosis of cancer was feared because of the perception that it inevitably led to death. Now, owing to a combination of early detection and improved treatments, death rates due to cancer in the United States and other developed countries continue to fall [1]. However, these falling death rates, combined with an ageing population, mean that there are increasing numbers of cancer survivors who must adjust to their diagnoses, residual symptoms and side effects of treatment [2]. Many who have survived cancer report positive changes in their lives; however, resentment is also experienced, especially amongst those with physical deformities, health problems resulting from cancer and its treatment, residual pain and social isolation. All of these factors can adversely affect quality of life (QoL) more than 15 years beyond the initial diagnosis [3]. Despite these burdens, at a systems level, there remain gaps in survivorship care [4]. In many settings, there is not a comprehensive approach to the needs of cancer survivors, with follow-up focusing on surveillance rather than health promotion.

Among all cancers, few pose more challenges for survivors than head and neck (H&N) cancers. H&N cancers include cancers of the tongue; mouth; salivary glands; pharynx; oro-, hypo- and nasopharynx; nasal cavities; middle ear; sinuses; and larynx. Treatments can involve extensive surgery, which may result in marked disfigurement, especially if treatments include extensive neck dissection or removal of part of the maxilla or mandible. Some patients require exenteration of an eye, which has obvious implications for their self-esteem, functional ability and social relationships. It is unsurprising, then, that up to 75% of H&N cancer patients treated surgically have body image concerns [5]. In addition, the toxicities of concurrent chemotherapy and radiotherapy, which include haematological toxicity, desquamation, mucositis, pain, dysphagia and trismus, lead to delays in treatment in almost half of these patients [6]. Even after completion of treatment, these patients struggle with residual problems, such as reduced shoulder movement and shoulder and neck pain, all of which adversely affect their QoL [7].

Authors of a recent review reported that residual concerns at 12 months following completion of treatment included deterioration in physical functioning, xerostomia and sticky saliva [8]. Difficulty with saliva and swallowing can lead to concerns about social eating [9]. This has the potential to decrease social activities and erode relationships, which are predictive of anxiety in cancer survivors [10,11]. Fatigue further affects one-third of patients 12 months after completion of treatment, whilst appetite loss affects 36% [12]. Thus it is unsurprising that 35% of patients with H&N cancer experience clinical depression [13]. Pain, fatigue and depression persist in many patients [10], indicating the need for preemptive interventions rather than expectant watchfulness. Highlighting the iterative nature of these psychosocial issues is that heavy alcohol use, which is a risk factor for the development of H&N cancer [14], has been reported in up to one-third of patients with H&N cancer [15]. Stigma regarding disfigurement and guilt about one’s possible personal contribution towards the development of the cancer (through alcohol abuse or smoking) also adversely affect psychosocial adjustment [16].

The emergence of a new patient population with H&N cancer, those affected by human papillomavirus (HPV), is of particular concern. These patients are often younger, which is a risk factor for increased psychosocial distress [17]. However, to date, there has been a paucity of research examining the psychosocial impact of cancer in this population. Studies in which researchers have explored stigma about HPV have been confined to the association with cervical cancer and have revealed a greater level of stigma for males than for females [18]. Research in which investigators have assessed interventions to assist patients in coping with the stigma related to HPV in general is limited, although preliminary evidence suggests that educational messages may alter perceptions [19]. This patient population is highly vulnerable because of their younger age, the lack of research designed to define their specific needs and the absence of evidence to guide optimal interventions to promote adjustment.

### Interventions to assist head and neck cancer survivors

Given the complex, ongoing problems experienced by patients treated for H&N cancer, supportive interventions are vital for this population. However, compared with cancers such as breast and prostate cancer, H&N cancer has not received widespread attention, nor have H&N cancer patients had ready access to tailored psychosocial interventions or support groups. The authors of a review of interventions designed specifically for H&N cancer patients described some evidence of benefit due to psychoeducational initiatives [20]; however, few robust studies in this population have been conducted to date. In the United Kingdom, pilot testing of a psychoeducational intervention for patients during treatment demonstrated improvement in patient knowledge and body image [21]. Improvements in mood and QoL were also achieved through a psychoeducational intervention in one trial [22]; however, the trial was not randomised. Widespread implementation of the type of intervention used in that trial is likely to be limited by its therapist-intensive nature, as it requires four sessions, each lasting 90 minutes.

Our team developed a resource for patients who had completed treatment for H&N cancer. The resource, “Facing the Future: Living with Confidence after Treatment for Head and Neck Cancer,” is based on evidence about issues concerning patients treated for H&N cancer. These issues include physical changes, work, day-to-day tasks, interpersonal relationships and social functioning [23]. Members of the Royal Brisbane and Women’s Hospital (RBWH) multidisciplinary Ambulatory Care Enhancement Team (ACET) collaborated to provide information and practical recommendations about strategies patients could use to respond to these and other problems. An initial draft of the resource was prepared and critically reviewed by members of a local H&N cancer support group. Consumer feedback was incorporated into the draft, and the resource was further refined before a second round of consultation with members of the H&N cancer support group and the ACET team, after which the final version was prepared.

The 61-page resource guide was pilot-tested from late 2011 through early 2012 with 18 patients who had completed treatment for H&N cancer at RBWH. The positive endorsement of the content and information contained in the resource did not translate to reported changes in behaviour or levels or confidence. Only 39% of respondents said that having this resource would increase their confidence about coping after treatment “a lot”, and 39% said it would increase their confidence about coping either “not at all” or “a little”. Furthermore, no respondents indicated that they had made “a lot” or “an extreme amount” of change in their routines or had started new activities since reading the guide, and 22% had made “a fair bit” of change. The remainder had made only a little change, and 22% had made no changes at all. These pilot data highlight the limited impact of the provision of information alone in facilitating behavioural changes that have the potential to improve H&N patients’ QoL.

In contrast, emerging evidence from the field of chronic disease management demonstrates that an active collaborative approach to problem definition, in conjunction with the development of self-management strategies, leads to improved coping with symptoms [24,25]. In a pilot study of patients who had completed treatment for breast and colorectal cancer, Yates *et al*. [26] utilised this chronic disease self-management approach to develop a survivorship care plan focused on developing patients’ self-management capacity. There were significant differences between the control and intervention groups between the baseline and 6-month follow-up assessments. Compared with the control group, patients who received the self-management intervention had significantly greater improvement over time in their functional well-being and self-efficacy in maintaining relationships.

### Rationale for this study

The development of evidence-based models of cancer survivorship care remains important, especially for disadvantaged or marginalised patient populations [27]. In this study, we aim to improve the QoL of patients treated for H&N cancer through the H&N Cancer Survivor Self-management Care Plan (HNCP), which is based on the model for survivorship care plans advocated by the US Institute of Medicine [4] and informed by the principles contained in the chronic condition and self-management model published by the Australian Government Department of Health and Ageing [24,25]. Not a generic intervention, the will be an individualised management plan for each patient, in line with emerging evidence about the benefits of a prioritised care plan [28] that is explicitly aligned with the needs of individual patients [29].

Promotion of self-efficacy [30] is the fundamental theoretical platform of this intervention. *Self-efficacy* refers to an individual’s beliefs regarding his or her capacity to respond to challenges. Self-efficacy is postulated to predict (1) whether the individual will initiate a response to challenges, (2) how hard the patient will work at those challenges and (3) the extent to which the patient will persist despite adversity or setbacks. This model is ideally suited to developing self-management behaviours in patients treated for H&N cancer because the constellation of residual symptoms they experience may feel overwhelming, thereby undermining (1) their optimism about improving their health and (2) their beliefs about their ability to implement self-care actions that can effectively address their health concerns. An intervention targeting self-efficacy is likely to lead to sustained improvements in self-management of ongoing health concerns, with the improvements lasting beyond the duration of the intervention. Table 1 lists strategies that are used to promote self-efficacy.

**Table 1:** Techniques designed to promote self-efficacy

**Techniques**	**Methods**
Performance accomplishment	In this study, participants will be assisted to achieve incremental mastery over manageable tasks, which could in turn increase their willingness to engage in other self-care behaviours.
Vicarious experience	Participants in this study will observe the nurse modelling skills so that the can learn the steps required to complete a specific task.
Verbal persuasion	The nurse will build the patient’s confidence and provide encouragement, as there is evidence that people who are persuaded that they possess the requisite skills to master a challenge are more likely to apply greater effort than those who receive information alone, especially if the information comes from a credible source.
Attention to physiological states	Anxiety affects patients’ attitudes about their ability to deal with problems and reduces performance, so relaxation and other techniques will be used to reduce physiological arousal.

## Methods/Design

### Study aims and hypotheses

Our primary aim in this study is to evaluate the effectiveness of a focused intervention (the HNCP) in improving the QoL of H&N cancer survivors. The HNCP will be developed for each patient in collaboration with an oncology nurse who has participated in focused training in developing an individual’s self-management capacity and will receive clinical supervision during the delivery of the intervention.

Our secondary aims in the study are to evaluate (1) the impact of the HNCP on patient self-efficacy, mood and ability to engage in self-management behaviours that promote optimal health and well-being, (2) the cost impact on the health system of using this model of care in routine clinical practice and (3) the feasibility of integration of the HNCP into routine clinical practice, including identification of systems and patient barriers through interviews with participating nurses, nursing and medical directors of clinics, and patients.

We hypothesise that (1) at 6 months following the intervention, patients who receive the HNCP will have significantly improved QoL compared with patients who do not receive the HNCP, (2) the HNCP will be highly acceptable to patients and (3) at 6 months following the intervention, patients who receive the HNCP will be using significantly fewer health-care resources than patients who do not receive the HNCP.

### Interventions

The study design is a prospective randomised controlled trial (RCT) of the HNCP intervention using pre- and postintervention assessments. The study will be conducted in the cancer services of two large tertiary referral centres that provide multidisciplinary care for patients with H&N cancer. Oncology nurses at both centres will be recruited to participate in focused training in order to be able to deliver the HNCP. Once a sufficient number of nurses are trained (approximately 15 nurses in total patients will be recruited. The central project manager will use a computer-generated list of random numbers to allocate recruited patients to one of the three study arms that are described in the subsections below.

#### Usual care

Patients in the usual care arm will continue to receive standard care and other usual supportive care measures, including medical treatments and health-care appointments. These patients will not have contact with the trained nurses other than in the course of routine clinical care.

#### Information only

In addition to usual care, patients in this group will receive a copy of “Facing the Future: Living with Confidence after Treatment for Head and Neck Cancer”. The central project manager will forward copies of this resource guide directly to patients randomised to this arm of the study. These patients will not have contact with the trained nurses other than in the course of routine clinical care.

#### Intervention

Each patient randomised to the intervention arm of the study will receive an individualised HNCP within 1 month of completion of treatment. The HNCP will be developed during a face-to-face supportive and educational session between the patient and a trained nurse. The session will last up to 60 minutes and will be focused on developing the patient’s self-efficacy to manage identified health concerns. During the consultation, the patient and nurse will collaborate to define problems of concern to the patient and develop strategies targeted to address these concerns through practical goal-setting and planning. Information will be provided about symptom management, and strategies to promote behaviour change will also be discussed (for example, in relation to smoking). The HNCP will define follow-up and engagement with health-care systems and sources of community and social support [31]. Patients will also receive a copy of the resource “Facing the Future: Living with Confidence after Treatment for Head and Neck Cancer” and continue to receive usual care. To facilitate consistent, ongoing support, a copy of the HNCP will be sent to the patient’s general practitioner. A summary of the three study arms is presented in Table 2.

**Table 2:** Differences in conditions between the three arms of this study

	**Study conditions**		
**Study arms**	**Individualised H&N cancer survivorship self- management care plan (HNCP)**	**Receive a copy of the resource “facing the future: living with confidence after treatment for head and neck cancer”**	**Continue to receive standard care and other usual supportive care measures, including medical treatments and health-care appointments**
HNCP (intervention)	√	√	√
Information	*	√	√
Usual care	*	*	√

√ Intervention component implemented.

*Intervention component not implemented.

### Participants

#### Oncology nurses

Oncology nurses currently working at either cancer service will be eligible to participate if they (1) are registered nurses, (2) have at least 12 months of clinical experience in oncology, (3) are currently engaged in patient contact for at least 6 hours per week, (4) can commit to undertake the necessary training and (5) work in a setting in which they can deliver the intervention (that is, the HNCP). Those nurses who anticipate leaving their current work setting within the next 12 months or taking a period of extended leave during the conduct of the study will not be eligible to participate. Informed consent will be obtained from all nurses recruited to participate in the study.

The recruited oncology nurses will undergo extensive manual-based training to develop advanced knowledge and skills in developing individualised self-management care plans for patients, specifically with respect to the following core concepts: (1) health promotion approaches, (2) assessment of health risk factors, (3) communication skills, (4) assessment of self-management capacity, (5) collaborative care planning, (6) use of peer support, cultural awareness, (7) psychosocial assessment and support, (8) motivational interviewing, (9) collaborative problem definition, (10) goal-setting, (11) action planning and (12) structured problem-solving [24]. After they have worked through the training manual, the nurses will participate in skill development conducted face-to-face by JT, PY and ALM. Decision support tools will provide a framework for the nurses to tailor the HNCP to the specific needs of each patient, and, whilst delivering the intervention, the nurses will participate in fortnightly clinical supervision. Clinical supervision will be conducted in a group format facilitated by JT, PY and ALM, who have extensive experience in supervision. During the supervision sessions, participating nurses will present an overview of the HNCPs that they have developed and will be assisted to devise strategies for responding to any challenges they experience. We aim to train 15 oncology nurses so that all will have the opportunity to deliver the HNCP on several occasions, but without posing a burden in terms of their routine clinical roles.

#### Patients

The patients eligible for this study must have received treatment for H&N cancer at one of the two cancer services. The inclusion criteria are (1) age 18 years or older; (2) completion of a defined treatment protocol for cancer of the tongue; mouth; salivary glands; pharynx; oro-, hypo- nd/or nasopharynx; nasal cavities; middle ear; sinuses; or larynx or completion of a defined treatment protocol for nonmelanoma skin cancers of the head and neck requiring treatment known to cause toxicity (for example, any one or combination of surgery, radiotherapy or chemotherapy); and (3) possess physical, cognitive and mental status enabling participation in the study. Exclusion criteria are (1) inability to speak and read English; (2) currently receiving low-toxicity treatment for H&N cancer (for example, laser therapy alone); (3) the presence of severe mental, cognitive or physical conditions that would limit the patient’s ability to participate in the study or that require regular ongoing specialist treatment; and (4) advanced disease if life expectancy is expected to be less than 6 months. Informed consent to participate will be obtained from all patients recruited into the study.

There is insufficient evidence from other RCTs on which to base calculation of an adequate sample size for assessing a psychosocial intervention for H&N cancer patients. There are also limited published data about meaningful changes in scores on the Functional Assessment of Cancer Therapy–Head and Neck (FACT–H&N) [32], although this QoL instrument is deemed the most appropriate measurement tool for capturing the unique concerns of patients with H&N cancer [33]. Hence, for this preliminary study, the sample size of 40 patients per group (a total of 120 patients) is based on our capacity to enrol and retain patients, and to conduct the interventions and follow-up, within the time frame for which the study is funded [34]. This study is not powered for hypothesis testing, but it will allow us to determine which changes in the QoL measurement are meaningful to patients and will assist us in calculating the sample size for a subsequent, larger, multisite RCT in which a formal economic cost-effectiveness study can be included.

#### Clinical directors and managers

We will provide information about the study to clinical directors and managers at baseline and aim to recruit all relevant managers at each site for interviews to be conducted upon completion of the study.

### Study integrity

Multisite ethical approval for both sites involved in recruitment of participants into this study has been obtained from the Human Research Ethics Committee of RBWH (HREC/13/QRBW/94, dated 24 April 2013), and administrative ethical approval has been obtained from the human research ethics committees of the University of Queensland (Medical Research Ethics Committee approval 2013000654, dated 24 May 2013) and the Queensland University of Technology (approval 1300000640, dated 11 October 2013). The study will be conducted in accordance with the ethical standards of the responsible institutional human ethics committees and the Declaration of Helsinki (1983 revision). The study will be conducted in adherence to the CONSORT (Consolidated Standards of Reporting Trials) statement on randomised trials [35] and will be carried out concurrently at the two cancer services. Every consecutive patient attending the outpatient H&N cancer clinic at each cancer service will be screened for eligibility by a trained research assistant and enrolled if eligible and consenting. Careful documentation of outpatient attendance, subsequent enrolments and consent interviews will ensure that no patient is “selected” for inclusion or exclusion from the study.

The central project manager will allocate patients to one of the three study arms using a computer-generated list of random numbers. The manager will notify trained nurses, who will then contact those patients who have been randomised to receive the intervention. Fidelity to the treatment protocol will be ensured through rigorous training of nurses by means of a standardised manual, and skills development will be facilitated by JT, PY and ALM. In addition, nurses will participate in fortnightly supervision whilst delivering the interventions. At least one session conducted by a nurse each month will be audio-recorded to assess compliance with the study protocol and to provide feedback to the nurse. The central project manager or his or her delegate will forward copies of the resource guide “Facing the Future: Living with Confidence after Treatment for Head and Neck Cancer” directly to patients randomised to the information-only arm of the study.

Research staff involved in pre- and postintervention assessments will be blinded to the allocation status of the patient, as will clinical staff. If group allocation is inadvertently revealed to research staff by patients during follow-up assessments, this information will be recorded and analysis will be undertaken to assess for any effects.

No participating oncology nurses will be randomised. All will receive the training and will have an opportunity to deliver the HNCP to patients who have been randomised to receive the intervention.

### Measures

#### Oncology nurses

At recruitment, participating nurses will be asked to provide demographic information and details regarding their clinical experience and specific training in psychooncology. Upon conclusion of the study, the nurses will be asked to participate in a semistructured interview designed to elicit their perceptions of participation in the study as well as enablers and barriers to implementation of this model of care in routine clinical practice.

#### Patient measures

Patient measures will be completed at baseline and at 3-month and 6-month follow-up appointments.

### Clinical and demographic characteristics

At baseline, structured templates will be used to record age, marital status, education, tumour site, location and extent of metastases and anticancer and supportive therapies. Changes in clinical status and subsequent receipt of any anticancer treatment (surgery, chemotherapy and/or radiotherapy) and supportive treatments will be recorded at other time points to enable comparison of the intervention (the HNCP) with the information-only and usual-care groups on key clinical and treatment variables that may influence the effectiveness of the intervention or the outcomes of interest in this study.

### Primary outcome

QoL will be assessed using the FACT–H&N instrument [33]. The original FACT–General was developed and validated more than 10 years ago [36] and has been used in hundreds of studies worldwide. It is a self-report instrument consisting of 27 core items designed to assess function in 4 domains: (1) physical well-being, (2) social/family, (3) emotional well-being and (4) functional well-being. The FACT–H&N instrument includes an additional 12 items used to assess H&N cancer–related symptoms, which capture the unique concerns of this patient population that are not obtained with more generic measures [33]. It has been demonstrated to be a reliable and valid measure in this patient population [37]. Items are rated on a 0 to 4 Likert scale, then combined to produce subscale scores for each domain as well as a global QoL score.

### Secondary outcomes

#### Self-efficacy

The Cancer Behaviour Inventory version 2.0 [38] is a measure of self-efficacy for coping with cancer and includes a stress management scale. It consists of 33 items that are used to measure function in seven domains: (1) maintaining activity and independence, (2) seeking and understanding medical information, (3) managing stress, (4) coping with treatment-related side effects, (5) accepting cancer and maintaining a positive attitude, (6) regulating affect and (7) seeking support. This instrument is considered to be reliable and valid as well as sensitive to change over time [38].

#### Mood

The Hospital Anxiety and Depression Scale [39] is a 14-item scale that has good reliability and validity. It has been used extensively in studies of cancer patients. Cutoff scores of 22 and above represent severe disorder, and scores less than 8 represent no disorder.

#### Generic quality of life

The European Quality of Life Questionnaire (EuroQol) 5-level version (EQ-5D) [40] is a preference-based utility instrument that will be used for the economic analysis. The EQ-5D has five dimensions covering multiple aspects of QoL. It is a validated and reliable tool for utility measurement in economic studies. An algorithm has recently been developed for Australian populations within economic studies in Australia [41].

### Process measures

At the conclusion of the study, nurses will be asked a structured series of questions designed to assess perceived enablers and barriers to delivery of the HNCP in clinical practice. Similar information will be obtained from medical and nursing directors at the clinics. In addition, upon the completion of the intervention, patients in the intervention group will be asked to respond to a series of questions related to their perceptions of the effectiveness of the intervention sessions and their satisfaction with them. They will also be asked about factors that facilitated or impeded their ability to participate in the intervention and initiate behaviour changes.

### Economic study

The economic study will be undertaken from a health-care provider’s perspective (public and private hospitals) and will involve assessment of (1) intervention resources by identifying, quantifying and valuing resources using standard methods [42] and (2) utilisation and cost of doctor visits and medications for all participants over the course of a 12-month period covering 3 months preintervention, 6 months during the intervention and 3 months postintervention completion.

Investigators will maintain detailed records of direct costs of delivering the intervention (staffing, training, telephone costs, office consumables and printing and production of educational materials) for the duration of the study. Resources will be valued using standardised national fee schedules, salary schedules for personnel and retail prices for other general consumables. To test the occurrence of the medical offset effect as a benefit of the intervention, administrative data on doctor visits and medications will be requested from Medicare Australia records.

### Statistical analyses

Analyses will be conducted using IBM SPSS version 21 software (IBM SPSS, Chicago, IL, USA). Descriptive statistics of patients’ demographic and clinical characteristics at baseline will be generated for each treatment group for the purpose of considering the success of the randomisation. Summary measures at time 1 (baseline), time 2 (3-month follow-up) and time 3 (6-month follow-up) will be calculated as means for continuous variables and as proportions for categorical measures, including 95% confidence intervals. Separate analyses will be conducted for each outcome variable. For continuous variables, the difference between groups in the change in mean scores over time will be evaluated using a generalized linear mixed effects model with all available data controlled for the potential cluster effect of different sites. Otherwise, logistic regression analysis will be conducted. A group-by-time interaction effect will be included in all models. All analyses will be conducted on an intention-to-treat basis.

### Qualitative data

Audio-recorded intervention sessions will be transcribed, and content will be analysed (1) to document the frequency with which various strategies were used by nurses and the perceived effectiveness and satisfaction with the strategies and (2) to identify common themes that reflect issues impacting the intervention, including situational, patient-related, nurse-related or intervention-related factors. This analysis will provide useful information regarding intervention processes whilst also enabling assessment of adherence to the intervention protocol.

### Economic analyses

Intervention resource costs will be aggregated across the groups to assess the incremental cost differences. We will also explore the incremental health benefits of the intervention (each of the arms separately) over and above what occurs with usual care as measured by the EQ-5D scores for suitability within a potential cost-effectiveness analysis. Medicare data on doctor visits and medication use (type, quantity and associated cost) will be analysed to obtain means and 95% confidence intervals using bootstrapping statistics [43] to account for the expected rightward-skewing nature of health-care resource use. Bootstrapped mean differences in health-care utilisation across the three groups will be assessed using Wald tests and calculated using Stata/SE 12 software (StataCorp, College Station, TX, USA).

## Discussion

Despite increasing acknowledgement of the needs of cancer survivors, many cancer services still do not offer focused interventions to promote their well-being. Currently, there is a paucity of research demonstrating effective models of survivorship care that are feasible to deliver in busy clinical services, which are often resource-poor.

This study is the first of its kind in Australia. It targets a patient population that faces the additive challenges of intensive treatments and severe side effects, often against a background of premorbid vulnerability. Despite documented high levels of depression, the lack of a political voice and advocacy has seen the needs of this cancer population neglected relative to those of patients with other cancers, such as breast cancer or, increasingly, prostate cancer.

Embedding the intervention into clinical care is likely not only to be acceptable to patients [44] but also to address the barriers of stigma and the burden of travel to receive assistance [45-47], because the intervention can be delivered to patients in rural and remote areas by appropriately trained local nurses. Furthermore, it has been proposed that supportive care interventions for people with cancer will produce downstream economic benefits in the form of reduced use of physicians’ services and medications due to the intervention’s successfully meeting the specific needs of patients [48]. However, as yet, there is no firm evidence to support this medical “cost-offset effect”.

If successful, this study will (1) provide evidence about a model of care structured to improve QoL in a cancer patient population with unique and complex needs, (2) add to the evidence base with respect to chronic disease self-management interventions and (3) provide a basis for a larger multisite study designed to examine the feasibility and cost-effectiveness of this comprehensive survivorship intervention in different treatment settings.

## Trial status

Fifteen oncology nurses have been recruited and completed training in order to be able to deliver the intervention. Eight patients have been recruited across both sites to date.

## Abbreviations

ACET: Ambulatory care and enhancement team; ACTRN: Australian Clinical Trial Registry number; ENHANCES: Enhancing Head and Neck Cancer Patients’ Experiences of Survivorship; EQ-5D: European Quality of Life Questionnaire (EuroQol) 5-level version; FACT–H&N: Functional Assessment of Cancer Therapy–Head and Neck; H&N: Head and neck; HNCP: Head and Neck Cancer Survivor Self-management Care Plan; HPV: Human papillomavirus; QoL: Quality of life RBWH, Royal Brisbane and Women’s Hospital; RCT: Randomised control trial.

## Competing interests

The authors declare that they have no competing interests.

## Authors’ contributions

All authors made substantial contributions to the conception or design of the work or the planned acquisition, analysis or interpretation of data, and were involved in drafting the work or revising it critically for important intellectual content. JT developed the study concept and aims and initiated the project. The intervention was based on previous work by PY. JT and AF developed the patient resource “Facing the Future: Living with Confidence after Treatment for Head and Neck Cancer”. LK, BB, DT, BH, ALM, CP, RJC, AF, MB and KC assisted in the refinement of the study concept and the development of the protocol. LGG devised and will complete the economic assessment. HS will maintain the database and conduct statistical analyses. JT and BB will implement the protocol and oversee the collection of data. JT, PY and ALM will provide oncology nurse training and supervision in consultation with other authors. JT was responsible for drafting the manuscript. All authors read and approved the final manuscript.
